# Evaluation and Analysis of Cement Raw Meal Homogenization Characteristics Based on Simulated Equipment Models

**DOI:** 10.3390/ma17122993

**Published:** 2024-06-18

**Authors:** Lianwei Cao, Yongmin Zhou

**Affiliations:** College of Materials Science and Engineering, Nanjing Tech University, South Puzhu Road No. 30, Nanjing 211816, China; 202161103071@njtech.edu.cn

**Keywords:** cement raw meal, production equipment, homogenization characteristics, simulation study

## Abstract

In recent years, the variability in the composition of cement raw materials has increasingly impacted the quality of cement products. However, there has been relatively little research on the homogenization effects of equipment in the cement production process. Existing studies mainly focus on the primary functions of equipment, such as the grinding efficiency of ball mills, the thermal decomposition in cyclone preheaters, and the thermal decomposition in rotary kilns. This study selected four typical pieces of equipment with significant homogenization functions for an in-depth investigation: ball mills, pneumatic homogenizing silos, cyclone preheaters, and rotary kilns. To assess the homogenization efficacy of each apparatus, scaled-down models of these devices were constructed and subjected to simulated experiments. To improve experimental efficiency and realistically simulate actual production conditions in a laboratory setting, this study used the uniformity of the electrical capacitance of mixed powders instead of compositional uniformity to analyze homogenization effects. The test material in the experiment consisted of a mixture of raw meal from a cement factory with a high dielectric constant and Fe_3_O_4_ powder. The parallel plate capacitance method was employed to ascertain the capacitance value of the mixed powder prior to and subsequent to treatment by each equipment model. The fluctuation of the input and output curves was analyzed, and the standard deviation (*S*), coefficient of variation (*R*), and homogenization multiplier (*H*) were calculated in order to evaluate the homogenization effect of each equipment model on the raw meal. The findings of the study indicated that the pneumatic homogenizer exhibited an exemplary homogenization effect, followed by the ball mill. For the ball mill, a higher proportion of small balls in the gradation can significantly enhance the homogenization effect without considering the grinding efficiency. The five-stage cyclone preheater also has a better homogenization effect, while the rotary kiln has a less significant homogenization effect on raw meal. Finally, the raw meal processed by each equipment model was used for clinker calcination and the preparation of cement mortar samples. After curing for three days, the compressive and flexural strengths of the samples were tested, thereby indirectly verifying the homogenization effect of each equipment model on the raw meal. This study helps to understand the homogenization process of raw materials by equipment in cement production and provides certain reference and data support for equipment selection, operation optimization, and quality control in the cement production process.

## 1. Introduction

With restrictions on mining, cement plants have begun to gradually adopt alternative raw materials, leading to increased volatility in their composition, which has a significant impact on the quality of cement products. The cement production process typically comprises three stages: raw meal preparation, clinker firing, and cement making. Ensuring the stability of raw material quality is crucial for maintaining the quality of the final product. The stability of raw meal quality is mainly affected by the stability of its composition, the more stable the composition, the better the quality of raw meal. Therefore, homogenization of raw materials and raw meal is needed to reduce the fluctuation of composition [1,2,3].

In the context of cement production, homogenization refers to the process of mixing and stirring raw materials or semi-finished products from different sources and compositions to achieve uniform composition. The objective is to ensure stable material quality in the subsequent process step, thereby guaranteeing the consistency of the final cement product quality. The homogenization process can be divided into two stages: raw material homogenization and raw meal homogenization. The homogenization of raw materials occurs during the stage of raw material handling, with the objective of equalizing the composition of raw materials such as limestone, clay, and iron ore. Pre-homogenization stockpiles are typically employed to process the raw materials, thereby reducing their compositional fluctuations and ensuring stable quality for the subsequent step of raw meal preparation [4,5]. Raw meal homogenization occurs during the stage of raw meal preparation and involves a series of steps, including crushing, mixing, and grinding. By continuously mixing during storage and transport, the chemical composition of the raw meal is made uniform, ensuring consistent reactions during calcination and improving clinker quality. The principal techniques employed in the homogenization of raw meals include [6,7]:

(1) Gravity Homogenizing Silos: The utilization of gravity for layered stacking and mixing during discharge allows for the achievement of homogenization. The advantages of this system include its simple structure and ease of operation. However, it is limited by the material flowability and particle size distribution, which may result in incomplete homogenization;
(2) Mechanical Stirring Homogenizing Silos: This system employs mechanical stirring devices to achieve uniformity in the material. This method is effective in homogenizing materials with significant compositional fluctuations, but it is costly in terms of equipment, maintenance, and energy consumption;
(3) Pneumatic Homogenizing Silos: This system employs airflow to disperse and mix the material. This method has low energy consumption and good homogenization effects, rendering it suitable for powdery materials. However, it necessitates the use of complex equipment and strict adherence to air distribution and flow control requirements;
(4) Roller Press and Ball Mill Combined Homogenization: This system achieves even granularity and homogenization through the use of a roller press for pre-crushing and a ball mill for fine grinding. While the grinding efficiency and homogenization effect are high, the equipment investment and maintenance costs are considerable.

Homogenization is crucial in cement production for several reasons [8,9,10]:

(1) Reducing compositional fluctuations: The homogenization process can significantly reduce fluctuations in the composition of raw materials and raw meals, ensuring that the composition of the raw meal entering the calcination stage is stable. This stability guarantees the consistency of clinker and the final cement product quality, ensuring stable product quality;
(2) Improving kiln efficiency: Well-homogenized raw meal reacts more uniformly during calcination, significantly enhancing kiln operation efficiency and reducing energy consumption. It also minimizes the occurrence of kiln crusting and tuberculation, increasing the kiln’s operational rate and service life and thereby improving production efficiency;
(3) Reducing production costs: Homogenization can mitigate the adverse effects of compositional variations in raw materials and raw meals on product quality and reduce the incidence of rework, thereby reducing unnecessary waste in production and lowering overall production costs.

In recent years, there has been a paucity of studies investigating the impact of relevant equipment utilized in the cement production process on the homogenization of raw materials. While these equipment do exert a homogenizing effect on raw materials, the majority of existing studies have focused on the primary roles of the equipment, such as the grinding benefits of ball mills, the thermal decomposition of cyclone preheaters, and the thermal decomposition of rotary kilns, among others. Consequently, this study conducted experimental evaluations of the homogenization effects of four major pieces of cement production equipment (continuous ball mills, pneumatic homogenizing silos, cyclone preheaters, and rotary kilns) by designing and constructing equipment models. This research provides valuable references for equipment selection, operational optimization, and quality control in the cement production process. Additionally, the experimental data and calculated homogenization indicators offer data support for enterprises, making the comparison and optimization of equipment performance more intuitive and scientific.

## 2. Materials and Methods

### 2.1. Measurement Techniques and Material Selection

In the simulation experiments of the preparation and clinkering processes of cement raw meal, powder with a high dielectric constant is mixed into the raw meal powder. The uniformity of the capacitance values of the mixed powder is employed as a proxy for the uniformity of the composition, thus circumventing the time-consuming issue associated with X-ray fluorescence spectroscopy (XRF) measurements while ensuring that the experiments can be conducted in a laboratory environment. The details are as follows:

The capacitance measurement method employed in the simulation experiment adheres to the conventional parallel plate capacitance method. The parallel copper plate is connected to the digital bridge, which is subsequently connected to the computer. The measurement data are collected in real-time at the computer end. The digital bridge used was the 4080 model tester from Yisheng Victory Technology Co., Ltd. in Shenzhen, China. Due to the differing positions of the bridge connection line at the commencement of each experiment, a certain degree of discrepancy will be observed in the initial capacitance values. Consequently, the computer records the differences in capacitance. The raw meal powder from a cement factory in Zhejiang Province, China was selected as the experimental material, while the dopant material was chosen as Fe_3_O_4_ powder with a purity of ≥99.99%, a particle size of 100–200 mesh, and a high dielectric constant from Qinghe BoZuan Metal Material Co., Hebei Province, China, in order to facilitate the observation of the change in the capacitance value of the mixed powders.

### 2.2. Experimental Devices

**Figure 1:**
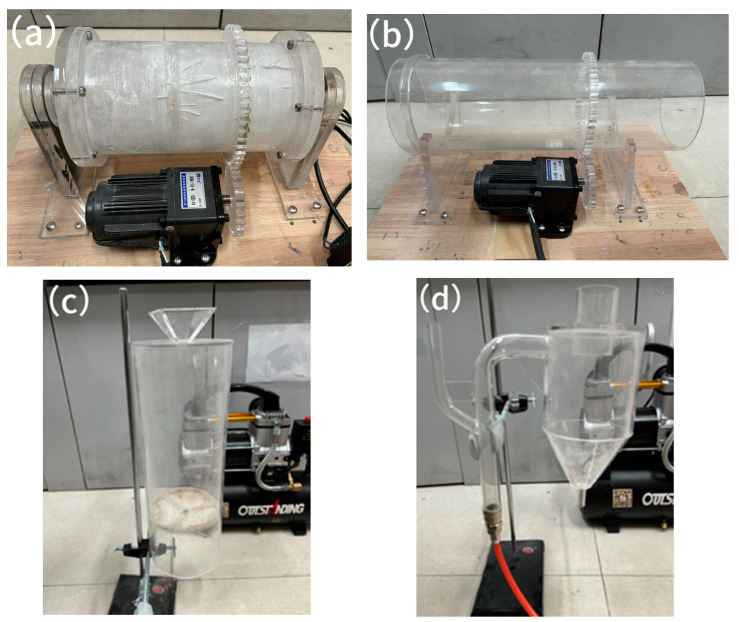
(**a**) The continuous ball mill model; (**b**) the rotary kiln model; (**c**) the pneumatic homogenizing silo model; (**d**) the one-stage cyclone preheater model.

In this study, each equipment model is separated and simulated separately, and the general basic devices used in each simulation mainly include the input pipe, the output pipe, and the disk feeder. The disk feeder is mainly used to control the flow of mixed powder into the equipment model by adjusting the rotational speed of the disk, the angle of the scraper plate, the reach length, and the distance from the input pipe. The designed and constructed models of each equipment are shown in Figure 1.

Each equipment model adopts acrylic plexiglass as the base material to ensure sufficient mechanical strength, while the movement of the powder in the model can be observed. The continuous ball mill model and the rotary kiln model use a motor with the power of 25 W and a rotational speed of 0~166 r/min as the power source, which rotates the cylinder through a gear drive, and the rotational speed range can be adjusted to 0–83 r/min. The pneumatic homogenizing silo and the one-stage cyclone preheater model use a small air compressor with the power of 1100 W and a pressure range of 0~0.7 MPa as the filling device, and the compressed air flow of the air compressor is used to mix the mixed powders. The air compressor was sourced from Aotus Industry and Trade Co., Ltd. in Taizhou, China, and the motor was sourced from Yixing Technology Co., Ltd. in Wenzhou, China. The relevant dimensions of each equipment model are as follows:

(1) The continuous ball mill model has a cylinder with an inner diameter of 100 mm, an effective length of 300 mm, and a thickness of 10 mm. The coarse grinding chamber has a length of 140 mm and a volume of about 1099.56 cm^3^, while the fine grinding chamber has a length of 160 mm and a volume of about 1256.63 cm^3^;
(2) The pneumatic homogenizing silo model has a cylinder with an inner diameter of 125 mm, a height of 400 mm, and a wall thickness of 5 mm. The discharge outlet diameter is 10 mm;
(3) The one-stage cyclone preheater model has a discharge tube with an inner diameter of 11 mm, heat exchange tubes with an inner diameter of 20 mm, a cyclone cylinder with an inner diameter of 100 mm, a height of 100 mm, and a discharge outlet diameter of 10 mm;
(4) The rotary kiln model has a cylindrical body with an inner diameter of 100 mm, a length of 300 mm, and a thickness of 5 mm.

### 2.3. Experimental Steps

The steps for each simulation experiment in this study are essentially the same. First, 1 kg of Fe_3_O_4_ powder is added to 2 kg of raw meal powder and then continuously poured into a vertical square tube. Once the powder accumulates to a certain height, the disk feeder is activated. The capacitance value of the mixed powder at the copper plate position is measured every 0.2 s for a total duration of 30 s, and the data are collected on a computer via remote communication. Meanwhile, the powder flows out through the input pipe to the disk feeder, where it enters the ball mill, pneumatic homogenizing silo, one-stage cyclone preheater, and rotary kiln models at a constant flow rate through the scraper of the disk feeder. Then, the mixed powder flows out from the respective equipment models into the output pipe. The measurement method at the output side is the same as that at the input side: when the powder accumulates to a certain height, the disk feeder is activated, and the capacitance value of the powder mixture at the copper plate position is measured every 0.2 s, again recorded for a total of 30 s, and the data are collected on a computer. To ensure the accuracy of the experimental results and to eliminate possible random errors in individual experiments, each simulation experiment was repeated three times. The homogenization effect of the powder during the accumulation process in the disc feeder is relatively low and can be considered negligible in comparison to the homogenization effects of other equipment.

In the simulation experiments on the continuous ball mill, glass balls were utilized as the grinding body, as the focus was on the impact of the agitation and mixing effect of the ball mill on the homogenization effect of raw materials, without consideration of the grinding benefit of the grinding body on the powder. Furthermore, three distinct gradations were evaluated to assess the influence of the ratio of large and small balls on the homogenization effect. The first gradation exhibited a higher proportion of large balls, the second gradation exhibited a balanced proportion of large and small balls, and the third gradation exhibited a higher proportion of small balls. The specific configurations are presented in Table 1.

For the pneumatic homogenizing silo simulation experiment, it is important to note that the mixed powder enters the silo through the central feed opening at the top via the scraper of the disk feeder at a constant flow rate, while the discharge valve at the center of the silo bottom is initially closed. When the powder has accumulated to about 70% of the silo height, the air compressor valve is activated to provide pneumatic agitation, and at the same time, the discharge valve is opened to allow the powder to flow under its own weight into the discharge port and further into the output pipe for subsequent measurements.

For the simulated experiments with the cyclone preheater, it is important to note that, prior to the powder entering the cyclone preheater, the air compressor must be activated and the air hose connected to the heat exchange duct to maintain an aerated state within the cyclone. The powder is then processed in the preheater model and exits through the lower discharge pipe into the output duct for subsequent measurement. In the context of cement production, a five-stage cyclone preheater system is typically employed. Consequently, in order to assess the homogenization effect of a five-stage cyclone preheater, the mixed powder processed by the single-stage cyclone preheater model is collected and re-injected into the feed inlet, repeating the process five times in order to simulate the operation of a five-stage cyclone preheater. Following the completion of the final processing stage, the parallel plate capacitor method is used for measurement.

**Figure 2:**
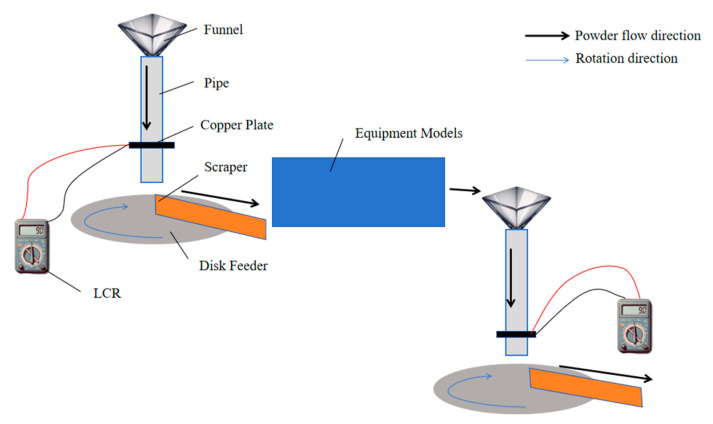
Schematic diagram of the experimental procedure.

The process parameters for the simulated experiments with each equipment model are shown in Table 2, and the experimental procedure is illustrated in Figure 2.

### 2.4. System Error Analysis

In this experiment, potential system errors may affect the reliability and accuracy of the results. To ensure the validity of the experimental results, it is necessary to identify and analyze these potential system errors. The following are the possible system errors and their impacts based on the experimental method used:

Firstly, in the capacitance measurement method, the connection method between the parallel plate capacitor and the digital bridge may introduce system errors. The position and connection method of the bridge wires may differ each time the experiment commences, resulting in variations in the initial capacitance value. To mitigate such errors, the experimental program is designed to collect the difference from the initial value and measure it three times, thereby improving measurement accuracy.

Secondly, the initial degree of mixing of the raw materials may also introduce errors. Inconsistencies in the degree of mixing when mixing Fe_3_O_4_ powder with raw meal may result in differences in the initial conditions between experiments. However, since the relationship between input and output was assessed between each group of experiments individually, rather than absolute homogeneity, differences in initial conditions had a relatively minor effect on the final homogenization effect.

Thirdly, the inconsistency in operating conditions of the model equipment represents another significant potential source of error. Fluctuations in the operating parameters (e.g., rotation speed, pressure, and inclination angle) of the ball mill, pneumatic homogenizing silo, cyclone preheater, and rotary kiln may result in discrepancies in experimental outcomes. Consequently, it is imperative to guarantee the stability and uniformity of the operational parameters throughout the experiment. For instance, the rotation speeds of the ball mill and rotary kiln should be regulated by controllers to ensure consistency. Similarly, the pressure of the air compressor should be maintained at a constant level for the homogenizing silo and cyclone preheater.

Finally, errors in data collection and processing may also be considered potential system errors. The response time of the data acquisition system may result in delays in data collection, which in turn may lead to inaccurate recording of capacitance values. The digital bridge utilized in this experiment is connected via USB, and the associated data transmission delays are minimal, typically within the millisecond range. Consequently, this error is relatively insignificant. Nevertheless, it is imperative to implement appropriate corrections and validations during the data processing stage.

### 2.5. Theoretical Analysis of Homogenization Effect

In the theoretical analysis of the effects of homogenization, three main indices are used: Standard Deviation (*S*), Coefficient of Variation (*R*), and Homogenization Factor (*H*). These indices comprehensively evaluate the homogeneity of powder mixtures and provide a scientific basis for optimizing the mixing process. The following is a detailed analysis of these three indicators [11,12]:

1. Standard Deviation (*S*)

Standard deviation measures the degree of dispersion among data in a dataset and is used to evaluate the consistency of component concentrations in powder mixtures. In this study, the standard deviation is used to quantify the variation in component concentrations in the mixture of Fe_3_O_4_ powder and raw meal powder. A lower standard deviation indicates that the concentrations of the components in the mixture are closer to each other, indicating better homogenization; a higher standard deviation indicates greater differences in component concentrations, indicating poor homogenization. The formula for calculating the standard deviation is shown below.

(1) $$S=\sqrt{\frac{{\left(X_{1}-\bar{x}\right)}^{2}+{\left(X_{2}-\bar{x}\right)}^{2}+\cdots +{\left(X_{N}-\bar{x}\right)}^{2}}{N-1}}$$

where $\bar{x}$ is the average capacitance value, *X_i_* is the capacitance value measured at each moment, *N* is the total number of measurements, and *S* is the standard deviation of capacitance values.

2. Coefficient of Variation (*R*)

The coefficient of variation, also known as the relative standard deviation, is the ratio of the standard deviation to the mean. It is used to describe the size of the standard deviation relative to the mean. This index provides a dimensionless measure of the homogeneity of mixtures, allowing the homogenizing effects of mixtures at different concentration levels to be compared. A lower coefficient of variation indicates that the components of the mixture are more consistent, while a higher coefficient indicates an uneven distribution of the components. The formula for calculating the coefficient of variation is shown below.

(2) $$R=\left(\frac{S}{\bar{x}}\right)\times 100\%$$

3. Homogenization Factor (*H*)

The homogenization factor is a measure of the improvement in the uniformity of the component distribution before and after mixing. This value is the ratio of the standard deviations of the component concentrations before and after mixing, used to quantify the effectiveness of the mixing process in enhancing the homogeneity of the mixture. Ideally, the higher the homogenization factor, the greater the mixing effect. In practical applications, pursuing a higher homogenization factor is an important goal in optimizing mixing processes. The formula for calculating the homogenization factor is shown below.

(3) $$H=\frac{S_{1}}{S_{2}}$$

The three indicators, obtained through precise measurement and calculation, can be employed to objectively evaluate the effectiveness of the powder mixing process. In this study, the parallel plate capacitor measurement technique was employed to monitor the capacitance values of mixed powders at the input and output in real time. The capacitance values were then employed to calculate a number of indicators, thus enabling an analysis of the homogenization effect of Fe_3_O_4_ and raw meal powders in each equipment model. Nevertheless, these indicators still have certain limitations [12,13]:

(1) Limitations of a single indicator: The homogenization coefficient is unable to provide a comprehensive assessment of the overall performance of the material due to its inability to reflect the uniformity of the chemical composition, physical and mechanical properties, and final product quality of the material;
(2) Ignoring local non-uniformity: The homogenization coefficient is a global indicator and may mask local non-uniformity. If the mixing effect is poor in certain areas, the homogenization coefficient may not fully reflect these local differences;
(3) Accuracy limitations of standard deviation: The standard deviation is influenced by the distribution of the data. In case the data distribution is uneven or there are outliers, the accuracy of the homogenization coefficient may be adversely affected;
(4) Dynamic changes: The flowability and mixing conditions of materials in the actual production process are subject to dynamic change. As a static indicator, the homogenization coefficient may not accurately reflect these changes in real-time.

To enhance the comprehensive evaluation of the powder mixing process, future research may consider integrating multiple indicators in conjunction with chemical composition analysis, physical and mechanical property testing, and other methodologies, in order to provide a more comprehensive evaluation. It should be noted that this study did not conduct the additional tests. Nevertheless, the provided methods and data analysis steps retain a certain value as a reference.

### 2.6. Compressive and Flexural Strength Testing

Theoretically, cement with a uniform raw meal composition exhibits more homogeneous hydration reactions and crystal structures, which reduces the possibility of incomplete reactions and defects, thus enhancing compressive and flexural strength. Consequently, in order to indirectly respond to the homogeneity of raw meal composition from the physical properties of cement, the raw materials obtained from the model processing of each equipment were calcined for clinker, and cement mortar specimens were made and tested for compressive and flexural strength.

Due to the high uniformity of raw meal composition used in previous capacitance experiments, the raw meal in this experiment was re-proportioned from various raw material powders. The composition ratios of the raw materials are shown in Table 3.

**Figure 3:**
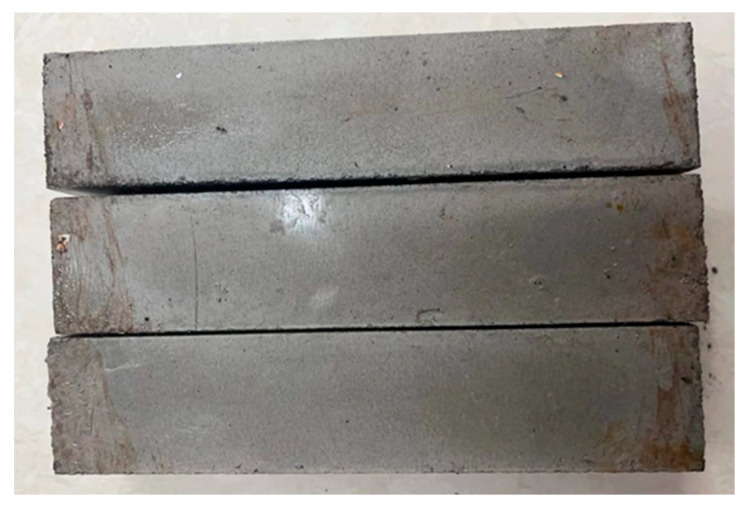
Some of the cement mortar samples.

The experimental procedures are as follows:

(1) Preparation of Clinker: Firstly, the raw meal was prepared according to the mixing ratios shown in Table 3. Subsequently, 1 kg of raw meal was added to each equipment model for treatment. The processed raw meal was then pressed into 15 disc-shaped samples with a diameter of 5 cm using a press at a pressure of 5 MPa, with each sample using 50 g of powder. The samples were then preheated at 950 °C for 30 min, followed by calcination at 1400 °C for 30 min to prepare the clinker;
(2) Preparation of Mortar Samples: The calcined clinker was powdered, and 3% gypsum by total weight was added. Subsequently, 450 g of cement powder, 1350 g of sand, and 225 mL of water were mixed thoroughly to obtain the mortar. The mixed mortar was then poured into rectangular molds with dimensions of 40 mm × 40 mm × 160 mm and compacted using a vibration table to ensure the absence of air bubbles and voids. The samples were cured in a humid environment at 20 ± 1 °C for 24 h. After 24 h, the samples were demolded and then placed in a curing chamber at 20 ± 1 °C with a relative humidity of no less than 90% for three days. Some samples are shown in Figure 3;
(3) Performance Testing: The cement mortar samples cured for three days were tested using a compressive strength testing machine and a flexural strength testing machine.

### 2.7. Repeatability of the Method

The research methods proposed in this article are characterized by good reproducibility, as evidenced by the following aspects:

(1) Detailed description of experimental equipment and operating parameters: The article provides a detailed description of the equipment models, operational parameters, and measurement methods employed in the experiments. This information provides sufficient guidance for other researchers to replicate the experiments under the same or similar conditions;
(2) Standardized measurement methods: Using capacitance measurement methods to evaluate homogenization effects is a simple and effective approach. Other researchers can follow the steps and methods provided in this article, using the same equipment and parameters for measurement, to ensure the comparability of experimental results;
(3) Data analysis methods: The statistical indicators used in the article, such as standard deviation, coefficient of variation, and homogenization factor, can quantify the homogenization effect. These indicators are universally applicable in different experiments and studies, allowing other researchers to use the same methods for data analysis to validate and extend the findings of this study.

## 3. Results

### 3.1. Curve Diagram Analysis

**Figure 4:**
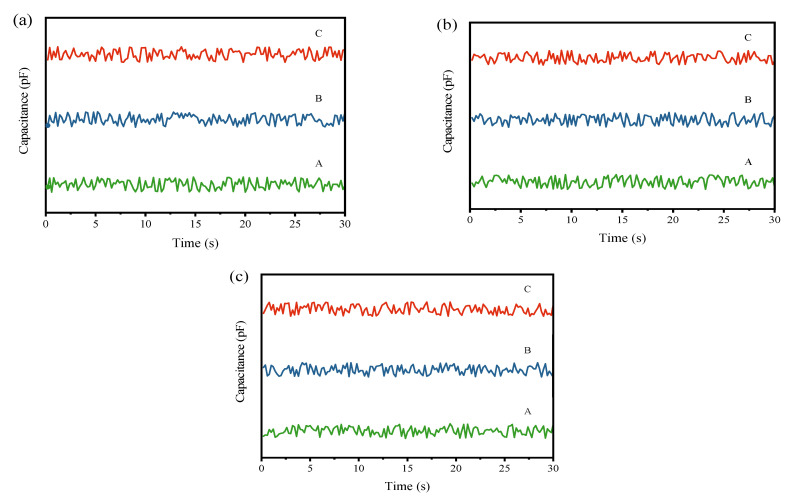
(**a**) Input capacitance variation curve of the ball mill simulation experiment under the first grading; (**b**) input capacitance variation curve of the ball mill simulation experiment under the second grading; (**c**) input capacitance variation curve of the ball mill simulation experiment under the third grading.

**Figure 5:**
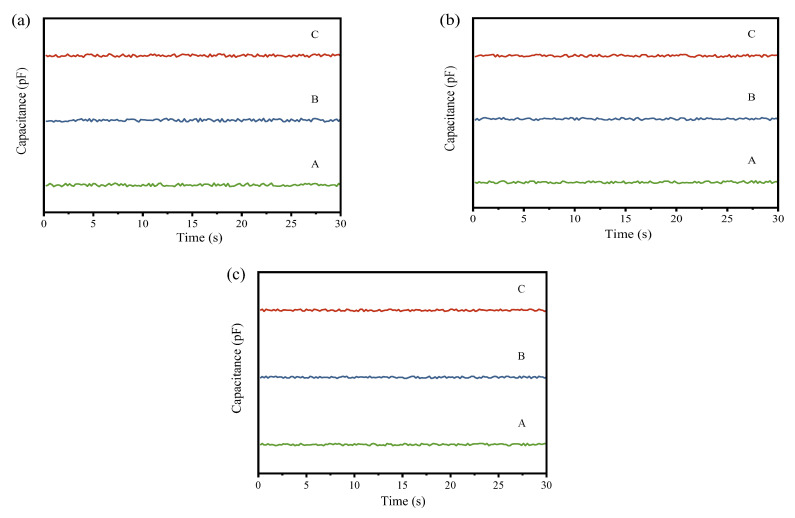
(**a**) Output capacitance variation curve of the ball mill simulation experiment under the first grading; (**b**) output capacitance variation curve of the ball mill simulation experiment under the second grading; (**c**) output capacitance variation curve of the ball mill simulation experiment under the third grading.

To visually observe the changes in powder mixing uniformity before and after processing by the continuous ball mill model with different gradings, the real-time capacitance values collected at the input and output ends were plotted into graphs. The input capacitance curve is depicted in Figure 4, and the output capacitance curve is illustrated in Figure 5.

In the Figure, A, B, and C represent the first, second, and third simulation experiments, respectively. The comparative experiments of the three gradings demonstrate that each group exhibits effective homogenization effects. As seen from the output curves in Figure 5, while there is no discernible difference between the three groups, the grading with a higher proportion of large balls exhibits a slight increase in fluctuation at the output compared to the other two groups. To obtain a more accurate assessment of the homogenization effect, further analysis is required using subsequent quantitative indicators.

**Figure 6:**
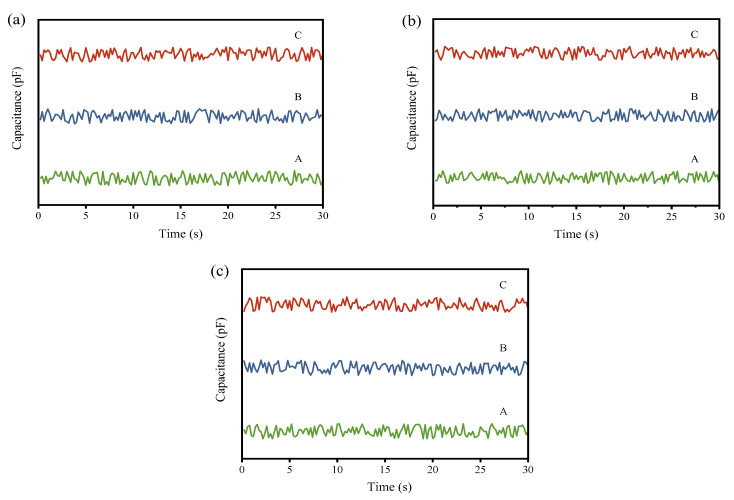
(**a**) Input capacitance curve for the homogenizing silo simulation experiment; (**b**) input capacitance curve for the cyclone preheater simulation experiment; (**c**) input capacitance curve for the rotary kiln simulation experiment.

**Figure 7:**
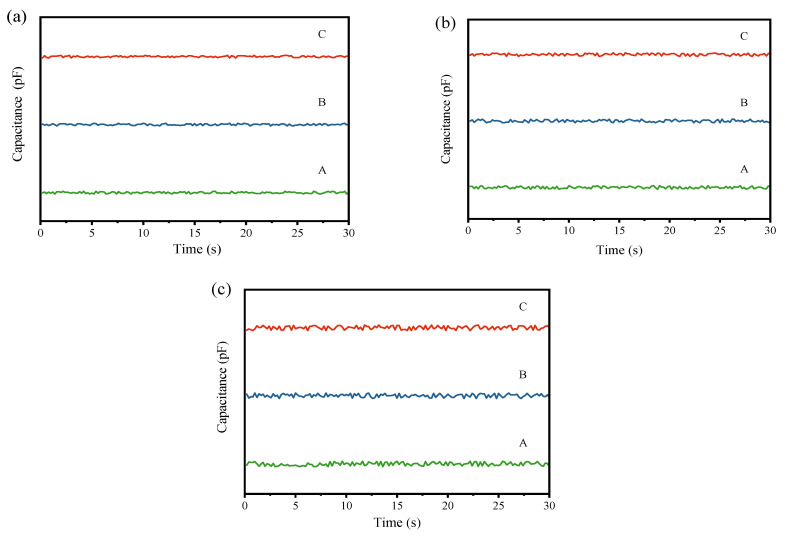
(**a**) Output capacitance curve for the homogenizing silo simulation experiment; (**b**) output capacitance curve for the cyclone preheater simulation experiment; (**c**) output capacitance curve for the rotary kiln simulation experiment.

Subsequently, to clearly observe the changes in the mixing uniformity of the mixed powder before and after processing by the pneumatic homogenizing silo, cyclone preheater, and rotary kiln model equipment, the real-time capacitance values collected at the input and output ends were plotted into graphs. The time-varying curves of the capacitance values at the input and output ends are presented in Figure 6 and Figure 7. In the figures, A, B, and C similarly represent the first, second, and third simulation experiments, respectively.

From the input capacitance variation curves in Figure 4 and Figure 6, it can be observed that in the three simulation experiments for each model, the capacitance value curves exhibit significant fluctuations, generally presenting an oscillating pattern. The peaks and troughs of the waveform indicate the local maxima and minima of the capacitance values, which correspond to instantaneous fluctuations in the material mixing state. These fluctuations may be attributed to the formation of distinct density regions or particle size distributions following the mixing of the raw meal and Fe_3_O_4_ powder.

In Figure 5 and Figure 7, the amplitude of fluctuations in the three curves is similar, and no extreme peaks or troughs are observed, indicating that the capacitance values of the mixed powder output in the three experiments have similar variability. The close proximity of the three curves to each other suggests high stability and reliability of the measurements.

From these curves, it can be analyzed that each equipment model indeed has a certain effect on the homogenization of the raw meal. The low fluctuation and stability of the output curves indicate that, although adding Fe_3_O_4_ powder initially increases the capacitance non-uniformity of the powder mixture, the final mixture shows better uniformity in capacitance values after processing. Comparing the output curves of each equipment model in Figure 5 and Figure 7, it can be observed that the ball mill model and pneumatic homogenizing silo exhibit the best homogenization effects, with minimal fluctuation amplitude; followed by the cyclone preheater; and the rotary kiln shows the weakest homogenization effect, with still a certain degree of fluctuation.

### 3.2. Equipment Repeatability

The curves plotted from the output data of the three sets of experiments for each model exhibit similar trends, indicating both the high stability and reliability of the measurements and the repeatability of the equipment.

### 3.3. Evaluation of Homogenization Effect

First, the input and output data obtained from the continuous ball mill model experiments under different gradings were substituted into Equations (1)–(3) to calculate the relevant homogenization indicators. The results of this calculation are shown in Table 4.

A comparison of the indicators obtained from the calculation revealed that the homogenization coefficient in the ball mill simulation experiments with a high percentage of large balls was between 4 and 5, while the homogenization coefficient with a high percentage of small balls was above 6. The reasons for this discrepancy were analyzed from the working principle as follows [14,15]:

(1) A high percentage of small balls can more effectively fill and cover the material surface, increasing the contact area with the material and thereby improving the grinding and homogenization effect. However, the impact force of small balls is relatively low, which may result in lower crushing efficiency, especially for larger or harder materials, where the grinding effect may not be ideal;
(2) In the case of a large proportion of large balls, due to the relatively small contact area between the large balls and the material, the grinding and homogenization effect is not as delicate as it would be with smaller balls. Additionally, the fine particles in the material may not sufficiently contact and grind due to the larger gaps between the large balls. However, large balls have a greater impact force, allowing them to break larger particles more effectively and improve crushing efficiency.

Consequently, it can be postulated that an increased number of small balls would facilitate the homogenization process, as they would be capable of grinding the material in a more uniform manner and increasing the contact area. In practical applications, however, it is often necessary to calculate the proportion of large and small balls based on the characteristics and production requirements of the material. This is achieved by utilizing the high impact force of large balls to break large particles and the high contact area of small balls to finely grind the material, thereby balancing the efficiency of crushing with the homogenization effect.

The following Table 5 presents the homogenization indicators calculated from the simulation experiment data of the homogenizing silo, cyclone preheater, and rotary kiln models.

Based on the calculation results in the table above, the following conclusions can be drawn:

(1) Standard Deviation Analysis: The standard deviation of the input capacitance values is greater than that of the output capacitance values, indicating that the processing by each model significantly reduces the variability in capacitance values;
(2) Coefficient of Variation Analysis: The coefficient of variation of the output capacitance values is significantly smaller than that of the input capacitance values, indicating that the output capacitance values are relatively more stable. The relative dispersion of the output capacitance values is considerably lower than that of the input capacitance values, indicating a significant improvement in uniformity;
(3) Homogenization Factor Analysis: In terms of the homogenization factor, the pneumatic homogenizing silo model demonstrates the most pronounced homogenization effect on the raw meal, with a homogenization factor exceeding 6. The continuous ball mill follows, with homogenization factors around 5 for all gradings. Next is the cyclone preheater model, and the rotary kiln model has the lowest homogenization effect on the raw meal, with a homogenization factor of only around 2.

Generally, a higher homogenization coefficient indicates a better mixing effect. However, different types of equipment and operating conditions may result in variations in the optimal homogenization coefficient [16,17]. According to industry standards, in modern cement production processes, common homogenizing equipment such as Continuous Flow Silos (CF Silos) and Multi-Material Flow Silos typically have homogenization coefficients ranging from 3 to 6. Under ideal conditions, this value can reach between 6 and 10 (Cement Indus Need). However, for the ball mill, cyclone preheater, and rotary kiln, homogenization is not their primary function. The main priority for these pieces of equipment is to ensure the primary processing of the raw meal, such as grinding, preheating, and calcining. Homogenization effects are considered secondary.

### 3.4. Compressive and Flexural Strength Analysis

The compressive and flexural strength of the cement mortar samples, prepared from the raw meal processed by each equipment model and tested after three days of curing, are presented in Table 6.

The data presented in Table 6 indicate that the test specimens prepared from the raw meal processed by the homogenization silo model exhibited the highest compressive and flexural strengths after three days of curing, reaching 27 MPa and 5 MPa, respectively. Excluding the effect of grinding, the raw meal processed by the ball mill under gradation conditions with a higher proportion of small balls resulted in cement with higher compressive and flexural strengths. In contrast, specimens prepared from raw meal processed by the cyclone preheater exhibited intermediate performance, while those prepared from raw meal processed by the rotary kiln exhibited the lowest strength. This indicates that each equipment model has a homogenization effect on the raw meal, with the homogenization silo exhibiting the most effective homogenization, followed by the ball mill, the cyclone preheater, and finally, the rotary kiln.

Generally, the more uniform the composition of the raw meal, the denser and more stable the structure of the resulting clinker, thereby enhancing the compressive and flexural strengths of the cement. This is primarily because the uniformity of the raw meal directly affects the crystallization degree and microstructure of the clinker. When the raw meal is uniform, the mineral phase distribution of the clinker formed during calcination is more homogeneous, and the bonding between the grains is tighter, resulting in cement with higher strength. Furthermore, a uniform raw meal composition assists in reducing local over-burning or under-burning during calcination, thereby enhancing the quality of the clinker and the mechanical properties of the cement [18,19].

From the perspective of the working principles involved, the differing homogenization levels of raw materials can be attributed to the varying working principles of each device.

The ball mill employs the grinding media within the rotating cylinder to repeatedly impact, grind, and frictionally deform the material, thereby enhancing the uniformity of the particle size distribution. The continuous tumbling and stirring facilitate increased contact and collision between particles, thereby improving the uniformity of the material [20,21].

The pneumatic homogenizing silo is designed to achieve efficient mixing of materials through the application of airflow. The apparatus employs compressed air to disperse the material, creating a suspended state that effectively mixes raw materials from disparate sources and compositions. The continuous airflow within the homogenizing silo facilitates the movement of the material, thereby increasing the collision and friction between particles, resulting in a more thorough and uniform mixing [22,23].

The high turbulence within the five-stage cyclone preheater facilitates complete mixing of the particles, disruption of agglomerates, and repeated mixing and separation of the raw material in multiple stages [24,25]. This process results in a more thorough homogenization of the raw meal.

However, the homogenization effect in the rotary kiln is not very significant due to the following reasons [26,27]:

(1) Material Layer Thickness: A thicker material layer reduces the effective contact and mixing between different regions inside the kiln, particularly affecting the exchange between the central and peripheral materials, limiting the achievement of homogeneous mixing;
(2) Differences in Material Flow ability: Differences in the physical properties of the material, such as particle size, density, and shape, result in varying flowability within the kiln. These differences hinder uniform material distribution and mixing;
(3) Limited Mechanical Stirring: Although the rotation of the kiln promotes axial and radial movement of the material, this stirring is relatively mild and insufficient to achieve the high-efficiency homogenization comparable to specialized mixing equipment.

## 4. Conclusions

This study evaluated the homogenization effect of four typical pieces of equipment (ball mill, pneumatic homogenizing silo, cyclone preheater, and rotary kiln) in the cement production process through simulated experiments and capacitance measurement techniques. The following findings were obtained:

(1) Simulation Experiments of the Ball Mill Under Different Grading Configurations: The experiments demonstrated that the grading with a high proportion of large balls exhibited a relatively lower homogenization effect on the raw meal, with a homogenization coefficient between 4 and 5. Conversely, the grading with a high proportion of small balls exhibited the most effective homogenization effect, with a homogenization coefficient exceeding 6. When the grinding efficiency is not considered, a higher proportion of small balls results in better homogenization of the raw meal under the same fill rate;
(2) Comparison of Equipment Model Simulations: The pneumatic homogenizing silo exhibited the most pronounced homogenization effect, with a homogenization coefficient exceeding 6. The material was effectively mixed through airflow, resulting in a notable reduction in material variability and an increased uniformity in the composition of the raw meal. The ball mill exhibited a homogenization effect that was second only to that of the pneumatic homogenizing silo, with a homogenization coefficient between 5 and 6. Subsequently, the cyclone preheater exhibited a homogenization coefficient of approximately 4, which achieved a certain degree of homogenization during preheating and decomposition. The rotary kiln exhibited the least effective homogenization, with a homogenization coefficient of only 2;
(3) In the strength testing experiments of cement mortar samples after three days of curing, it was found that the cement mortar samples prepared from the raw meal processed by the homogenization silo model exhibited relatively high compressive and flexural strengths, reaching approximately 26 MPa and 5 MPa. Following this, cement specimens prepared from raw meal processed by the ball mill model and in the grading condition with a higher percentage of small balls exhibited higher strengths than specimens prepared in other grading conditions. The cyclone preheater followed, and lastly, the samples prepared from the raw meal processed by the rotary kiln exhibited the lowest strength. This indirectly reflects that the homogenization effect of the homogenization silo on the raw meal is the best, followed by the ball mill, then the cyclone preheater, with the rotary kiln having the poorest homogenization effect.

In actual production processes, these pieces of equipment are typically operated in series. Following homogenization treatment by preceding equipment, the raw meal entering the rotary kiln is already largely uniform, thereby ensuring the stability of the calcination process and the quality of the clinker. The findings of this research provide valuable insights that can inform equipment selection, operational optimization, and quality control in cement production. These insights can assist enterprises in improving their production efficiency and product quality in practical applications. However, the models constructed in this study cannot fully simulate the operation of actual industrial equipment, so the relevant data can only serve as a reference. Future research may further introduce multiple evaluation indicators, combined with chemical composition analysis and physical–mechanical performance testing, to provide a more comprehensive assessment.

## Figures and Tables

**Table 1:** Grinding media gradation in the continuous ball mill model.

Number	Name	First Chamber			Second Chamber		
	Ball Diameter/mm	18	16	14	14	12	10
1	Proportion/(%)	45	30	25	40	30	30
	Weight/g	210.87	140.58	117.15	187.44	140.58	140.58
2	Proportion/(%)	25	45	30	25	40	35
	Weight/g	117.15	210.87	140.58	117.15	187.44	164.01
3	Proportion/(%)	15	45	40	15	40	45
	Weight/g	70.29	210.87	187.44	70.29	187.44	210.87

**Table 2:** Process parameters for each simulation experiment.

Equipment Model	Process Parameters
Continuous ball mill	Filling rate: 35%
	Rotational speed: 60 r/min
Homogenizing silo	Filling rate: 70%
	Air pressure: 0.6 MPa
One-stage cyclone preheater	Air pressure: 0.3 MPa
Rotary kiln	Inclination: 3%
	Rotational speed: 5 r/min

**Table 3:** The mixing ratios of the raw materials.

Name	Ratios	*w* (CaO)	*w* (SiO_2_)	*w* (Al_2_O_3_)	*w* (Fe_2_O_3_)
Limestone	84.09	46.61	6.28	1.17	0.39
Silica fume sludge	4.50	3.46	62.41	13.09	7.27
Bauxite	2.57	2.41	39.61	29.19	12.95
Iron powder	8.84	12.92	42.41	8.1	18.39
Fly ash	2.03	20.43	39.3	17.05	10.22

**Table 4:** Homogenization indicators of ball mill simulation experiments under different gradings.

Number	Number of Experiments	Name	Standard Deviation	Coefficient of Variation	Homogenization Factor
1	1	Input	0.0132	0.1166	4.88
		Output	0.0027	0.0244	
	2	Input	0.0129	0.1145	4.46
		Output	0.0029	0.0262	
	3	Input	0.0132	0.1168	4.61
		Output	0.0029	0.0258	
2	1	Input	0.0125	0.1121	5.23
		Output	0.0024	0.0224	
	2	Input	0.0135	0.1194	6.18
		Output	0.0022	0.0203	
	3	Input	0.0137	0.1207	5.91
		Output	0.0023	0.0217	
3	1	Input	0.0134	0.1198	7.18
		Output	0.0019	0.0166	
	2	Input	0.0128	0.1137	6.88
		Output	0.0019	0.0166	
	3	Input	0.0123	0.1079	6.56
		Output	0.0019	0.0167	

**Table 5:** Homogenization indicators for each simulation experiment.

Equipment Model	Number of Experiments	Name	Standard Deviation	Coefficient of Variation	Homogenization Factor
Homogenizing silo	1	Input	0.0133	0.1169	7.09
		Output	0.0019	0.0171	
	2	Input	0.0135	0.1195	6.87
		Output	0.0020	0.0179	
	3	Input	0.0138	0.1232	6.64
		Output	0.0021	0.0190	
Cyclone preheater	1	Input	0.0125	0.1107	1.88
		Output	0.0067	0.0596	
	2	Input	0.0014	0.1196	2.22
		Output	0.0061	0.0548	
	3	Input	0.0136	0.1214	2.05
		Output	0.0066	0.0593	
Rotary kiln	1	Input	0.0127	0.1111	2.19
		Output	0.0044	0.0383	
	2	Input	0.0125	0.1089	2.82
		Output	0.0044	0.0391	
	3	Input	0.0135	0.1193	3.15
		Output	0.0043	0.0376	

**Table 6:** Compressive and flexural strength of each cement mortar sample.

Name		Compressive Strength/MPa	Flexural Strength/MPa
Ball mill	gradation 1	18.6	2.6
		19.1	2.7
		19.4	3.5
	gradation 2	21.5	4.1
		23.1	3.9
		22.4	3.8
	gradation 3	25.2	4.5
		27.9	4.1
		27.4	4.8
Homogenizing silo		26.5	5.1
		27.1	5.5
		27.4	5.3
Cyclone preheater		20.5	4.5
		19.8	4.1
		21.5	4.6
Rotary kiln		15.6	2.5
		18.9	2.8
		14.8	2.7

## Data Availability

The original contributions presented in the study are included in the article, further inquiries can be directed to the corresponding author.

## References

[1] Elmrabet R., Hmidani Y., Mariouch R., Elharfi A., Elyoubi M.S. (2021). Influence of raw meal composition on clinker reactivity and cement proprieties. Mater. Today Proc..

[2] Diliberto C., Lecomte A., Mechling J.M., Lzoret L., Smith A. (2017). Valorisation of recycled concrete sands in cement raw meal for cement production. Mater. Struct..

[3] Mujumdar K.S., Ganesh K.V., Kulkarni S.B., Ranade V.V. (2007). Rotary Cement Kiln Simulator (ROCKS): Integrated modeling of pre-heater, calciner, kiln and clinker cooler. Chem. Eng. Sci..

[4] Bily P., Fladr J., Chylik R., Vrablik L., Hrbek V. (2019). The effect of cement replacement and homogenization procedure on concrete mechanical properties. Mag. Civ. Eng..

[5] Asachi M., Nourafkan E., Hassanpour A. (2018). A review of current techniques for the evaluation of powder mixing. Adv. Powder Technol..

[6] Kuchirka Y.M., Volodarsky E.T. (2019). Cement Quality Control by using Modern Radiation Methods of Chemical Analysis in the Process of its Production. Methods.

[7] Borshchev V.Y., Sukhorukova T.A. (2018). Current Status and Development of Production Technologies of Multicomponent Mixtures of Bulk Materials in Large Volumes. Adv. Mater. Technol..

[8] Hogg R. (2009). Mixing and Segregation in Powders: Evaluation, Mechanisms and Processes. KONA Powder Part. J..

[9] Xu J., Mukherjee D., Chang S.-K. (2018). Physicochemical properties and storage stability of soybean protein nanoemulsions prepared by ultra-high pressure homogenization. Food Chem..

[10] John J.P. (2020). Parametric studies of cement production processes. J. Energy.

[11] Wu H., Khan A.M. (2009). Quality-by-design (QbD): An integrated approach for evaluation of powder blending process kinetics and determination of powder blending end-point. J. Pharm. Sci..

[12] Makni F., Kchaou M., Cristol A.L., Elleuch R., Desplanques Y. (2017). A new method of mixing quality assessment for friction material constituents toward better mechanical properties. Powder Metall. Met. Ceram..

[13] Sridhar A., Liu L., Kouznetsova V.G., Geers M. (2018). Homogenized enriched continuum analysis of acoustic metamaterials with negative stiffness and double negative effects. J. Mech. Phys. Solids.

[14] Ramírez-Torres A., Penta R., Rodríguez-Ramos R., Merodio J., Sabina F.J., Bravo-Castillero J., Guinovart-Díaz R., Preziosi L., Grillo A. (2018). Three scales asymptotic homogenization and its application to layered hierarchical hard tissues. Int. J. Solids Struct..

[15] Hussain Z. (2021). Comparative study on improving the ball mill process parameters influencing on the synthesis of ultrafine silica sand: A Taguchi coupled optimization technique. Int. J. Precis. Eng. Manuf..

[16] Santosh T., Soni R.K., Eswaraiah C., Rao D.S., Venugopal R. (2020). Optimization of stirred mill parameters for fine grinding of PGE bearing chromite ore. Part. Sci. Technol..

[17] Mawassy N., Reda H., Ganghoffer J.F., Eremeyev V.A., Lakiss H. (2021). A variational approach of homogenization of piezoelectric composites towards piezoelectric and flexoelectric effective media. Int. J. Eng. Sci..

[18] Xiong G.-Q., Wang C., Zhou S., Zheng Y. (2022). Study on dispersion uniformity and performance improvement of steel fibre reinforced lightweight aggregate concrete by vibrational mixing. Case Stud. Constr. Mater..

[19] Li N., Ma B., Wang Y., Si W., Qin F. (2016). Influence analyses of mixing approaches on properties of conventional and interlocking-dense concrete. Constr. Build. Mater..

[20] Wang L., Li E.-L., Shen H., Zou R.-P., Yu A.-B., Zhou Z.-Y. (2020). Adhesion effects on spreading of metal powders in selective laser melting. Powder Technol..

[21] Jing G.-J., Ye Z.-M., Li C., Cui J., Wang S.-X., Cheng X. (2019). A ball milling strategy to disperse graphene oxide in cement composites. New Carbon Mater..

[22] Ghalandari V., Iranmanesh A. (2020). Energy and exergy analyses for a cement ball mill of a new generation cement plant and optimizing grinding process: A case study. Adv. Powder Technol..

[23] Xiang J.-S., McGlinchey D., Latham J.P. (2010). An investigation of segregation and mixing in dense phase pneumatic conveying. Granul. Matter.

[24] Ficici F., Ari V. (2013). Optimization of the preheater cyclone separators used in the cement industry. Int. J. Green Energy.

[25] Mirzaei M., Clausen S., Wu H., Nakhaei M., Zhou H., Jønck K., Jensen P.A., Lin W. (2023). Investigation of erosion in an industrial cyclone preheater by CFD simulations. Powder Technol..

[26] Aghdasinia H., Hosseini S.S., Hamedi J. (2021). Improvement of a cement rotary kiln performance using artificial neural network. J. Ambient Intell. Humaniz. Comput..

[27] Zhang T., Peng H., Chen R., Guo Y., Wang J., Chen X., Wei J., Yu Q. (2023). Enhancing simultaneous desulfurization and denitrification efficiency of cement kiln exhaust gas via co-catalytic Fenton oxidation process. J. Clean. Prod..

